# Sudden gains and early response in routine outpatient psychotherapy: a study protocol for predicting symptom change

**DOI:** 10.3389/fpsyg.2026.1897870

**Published:** 2026-10-19

**Authors:** L. Claus, F. Raulfs, J. Goltermann, M. Driessen, C. Steuwe

**Affiliations:** 1 Department of Psychiatry and Psychotherapy, Evangelisches Klinikum Bethel, Universitätsklinikum OWL of Bielefeld University, Bielefeld, Germany; 2 Medical School OWL, Bielefeld University, Bielefeld, Germany

**Keywords:** change attribution, common factors, mechanisms of change, routine outcome monitoring, sudden losses, symptom change

## Abstract

**Background:**

Sudden gains and early response have been established as reliable predictors of psychotherapy outcomes. However, there is still a lack of consistent findings regarding the predictors of these patterns of therapeutic change themselves. Thus far, demographic and clinical variables have provided only limited insight into why sudden gains and early response occur in some patients during therapy, are absent in others, or are reversed in the form of sudden losses.

**Methods:**

This prospective, transdiagnostic cohort study aims to identify therapy process variables and therapy-related patient variables that predict sudden gains/losses and early response in routine outpatient psychotherapy. Particular attention is given to common factors as mechanisms of change that operate across different therapeutic approaches, including therapeutic alliance, resource activation, problem activation, problem solving and motivational clarification. Treatment trajectories of 300 patients at a psychotherapy training center will be assessed from the initial trial sessions through session 27 using weekly therapy monitoring, including brief measures of symptom distress, daily functioning, and common factors. Follow-up data will be collected up to 3 months after treatment completion. Early response is defined as reliable symptom improvement by session 5, and sudden gains and sudden losses are determined according to established criteria. Regression analyses will be used to examine the contribution of therapy process variables, their temporal dynamics, and therapy-related patient variables to the prediction of sudden gains, sudden losses, and early response. In addition, the study will investigate whether reductions in symptom distress are accompanied by improvements in daily functioning. If sudden gains are detected by the monitoring system, qualitative telephone interviews will be conducted to explore patients’ subjective attributions regarding mechanisms of change.

**Discussion:**

Improved knowledge of mechanisms of change may help refine psychotherapy processes and support more targeted treatment planning. The methodological and practical challenges of the study are discussed.

**Clinical trial registration:**

https://www.drks.de/search/de/trial/DRKS00039264, identifier DRKS00039264.

## 1 Introduction

### 1.1 Background and rationale

Over the past decade, the importance of early treatment response has been examined in numerous, predominantly pharmacological studies, particularly in depression and obsessive-compulsive disorder. Meta-analytic findings indicate that symptom improvement of 20 to 50% within the first 4 weeks predicts later treatment response in treatment-resistant depression, whereas the absence of such early improvement substantially reduces the likelihood of subsequent remission (Olgiati et al., 2018; Wagner et al., 2017). In this context, the value of routine treatment monitoring is emphasized in the context of pharmacotherapy (Wagner et al., 2017). Studies of early response in psychotherapy or combined therapy have also received increasing attention in recent years, with research examining their significance for treatment outcomes (Rubel et al., 2015). Robust and replicated evidence shows that an early response to therapy is a reliable prognostic indicator of treatment success for depression (Schindler et al., 2013; Schlagert and Hiller, 2017; Persons and Thomas, 2019; Vittengl et al., 2019) and anxiety (Beard and Delgadillo, 2019; Bradford et al., 2011; Lutz et al., 2014; Vîslă et al., 2023). Particularly in the field of eating disorders, numerous studies—some of them with large effect sizes - demonstrate the importance of an early treatment response for a positive treatment outcome (Chang et al., 2021; Chen et al., 2017; Greenberg et al., 2022; Wales et al., 2016). Conversely, the absence of early response has been associated with lower symptom reduction, symptom deterioration, and reduced remission rates at the end of treatment (Schlagert and Hiller, 2015, 2017; Vittengl et al., 2019). The pattern of early response has been documented across a broad range of psychotherapy outcomes (Hunter et al., 2010), different therapeutic approaches (Crits-Christoph et al., 2001; Gunlicks-Stoessel and Mufson, 2011), and diagnostic groups (Bradford et al., 2011; Hayes et al., 2007; Lewis et al., 2012). Overall, the greatest change in symptom severity during psychotherapy occurs in the first phase of treatment (Rubel et al., 2015).

Despite extensive research, relatively little is known about the factors that determine early response (Bohart and Wade, 2013). Some studies have linked higher educational attainment, employment, and social support to an increased likelihood of early response, a tendency to avoid unpleasant feelings or states, chronicity of mental illness, and psychiatric comorbidity have been associated with a lower likelihood (Joska et al., 2023; Melchior et al., 2016). However, many studies have reported no associations with sociodemographic variables (e.g., age, gender) or clinical characteristics (e.g., symptom severity and functional level at the start of treatment, psychiatric comorbidity) (Bradford et al., 2011; Kelley et al., 2018; Kleinstäuber et al., 2017; Schlagert and Hiller, 2017; Vaz et al., 2014). Therapy and therapist related variables have been examined considerably less frequently, the variables examined to date (e.g., treatment expectations, therapists’ professional experience) have not demonstrated significant associations with early response (Haas et al., 2002; Lau et al., 2024). Findings, therefore, remain inconsistent: In some studies, baseline symptom severity has been associated with both posttreatment symptom severity and early response (Röhrig et al., 2024). First empirical indications suggest that the therapeutic relationship might be relevant for early response (Röhrig et al., 2024). These inconsistencies may reflect both methodological and clinical factors. Methodologically, studies differ considerably in their operationalization of early response (e.g., timing of assessment, percentage symptom reduction, or absolute cutoffs) and in sample size, leading to differences in statistical power to detect small to moderate effects. Clinically, associations may vary across therapeutic approaches, treatment settings and diagnostic groups.

In addition to early response, sudden gains, defined as large and rapid symptom improvements and typically following the criteria of Tang and DeRubeis (1999), have been identified as important predictors of treatment outcome and follow-up status (Aderka et al., 2012; Shalom and Aderka, 2020). Sudden Gains are most strongly associated with outcome in depressive and anxiety disorders (Shalom and Aderka, 2020), whereas they appear to play a minor role for treatment success in some other disorders, such as chronic pain (Bisby et al., 2024), obsessive-compulsive (Buchholz et al., 2019), and prolonged grief disorder (Lechner-Meichsner et al., 2025). In more intensive treatment settings, such as day treatment compared to outpatient therapy, sudden gains occur earlier and more frequently than in standard outpatient psychotherapy (Terrill et al., 2022).

As with early response, knowledge about patient characteristics and mechanisms that predict these sudden changes remain limited (Zilcha-Mano et al., 2019). Among the few identified predictors of sudden gains and correlates are, among others, the expression of “negative” emotions in the first trauma narrative among PTSD patients (Sloan et al., 2022), a higher overall quality of life (Lemmens et al., 2016), cognitive changes in critical sessions prior to the sudden gain (Lee et al., 2024; Norton et al., 2010; Tang et al., 2005), and prior outpatient psychotherapy (Otis et al., 2025), although the association with cognitive changes in particular is not consistently reported in the literature (Clerkin et al., 2008; Hofmann et al., 2006; Nogueira-Arjona et al., 2017). Symptom variability before and during therapy has also been associated with sudden gains; high variability is associated with a high probability of a sudden gain (Shalom et al., 2020). Additional findings regarding associations with sudden gains point to reductions in negative social cognitions and self-focused attention in social phobia (Thew et al., 2023; Yim et al., 2024), as well to the use of specific interventions such as exposure and response prevention (Bjureberg et al., 2020). The therapeutic alliance appears to be particularly important for the maintenance of sudden gains (Zilcha-Mano et al., 2019). At the same time, numerous studies have found no association between sudden gains and sociodemographic variables such as gender, age, education, and marital status (Krüger et al., 2014; Kuck et al., 2024; O’Mahen et al., 2021; Shalom et al., 2025) nor clinical characteristics such as comorbidities, duration of illness, symptom severity prior to treatment, or self-harm (Collins and Coles, 2017; Krüger et al., 2014; Shalom and Aderka, 2020; Singh et al., 2020). Evidence on therapy process variables is similarly sparse, and no associations have been found for frequency of therapist contact or session content (Bisby et al., 2024; Lemmens et al., 2021). Similar to the discussion of early response, the heterogeneous findings on sudden gains may partly reflect differences in therapeutic approaches, settings, diagnostic groups, and sample sizes across studies. However, the definition of sudden gains is considerably more uniform than that of early response, although variations in the scales used and the frequency of measurement still exist. These methodological differences may contribute to the remaining inconsistencies in the literature.

A small number of qualitative studies have examined subjective explanations for sudden gains. From a therapeutic perspective, retrospective explanations have included gaining insight, expressing previously concealed thoughts or feelings, and major external life events, although no consistent pattern has emerged (Davies et al., 2006). A qualitative analysis of patients’ statements during therapy revealed that increased active processing and coherence were evident prior to sudden gains (Adler et al., 2013). When asked directly, patients cited various factors as the cause of their sudden gain, including trying out new behaviors, changes in beliefs, and increased participation in leisure activities (Lemmens et al., 2021).

Since patient characteristics assessed at treatment onset have not consistently predicted early response or sudden gains, therapy process variables and transtheoretical common factors warrant particular attention. In this context, Grawe’s concept of common factors in psychotherapy is especially relevant, including the therapeutic relationship, motivational clarification, resource activation, problem actualization and problem solving (Grawe, 1997, 2005). There is substantial evidence that psychotherapy is specifically effective compared with placebo psychotherapy, and common factors are widely regarded as central contributors to this efficacy (Lambert, 2005; Wampold, 2015). Several studies have reported associations between a strong therapeutic relationship and early symptom improvement or early response (Flückiger et al., 2020; Röhrig et al., 2024). There is also preliminary evidence regarding the relevance of common factors for sudden gains, for example, when examining the therapeutic relationship (Zilcha-Mano et al., 2019) and insight or understanding (Goodridge and Hardy, 2009); although available findings remain limited. Importantly, such therapy process variables should not be understood as static characteristics but as dynamic processes that require repeated and ideally frequent assessment (Zilcha-Mano and Errázuriz, 2017). Obviously, in addition to common factors, other variables are also likely to influence treatment outcomes. For example, baseline symptom severity is associated with posttreatment symptom severity and symptom change trajectories during treatment, and there is compelling evidence that the strongest predictor of change in an outcome variable is the baseline value of that variable (Kamardina et al., 2026; Lambert et al., 2002).

It is currently nearly impossible for clinicians to make reliable predictions regarding the expected treatment course before therapy begins. Predicting early response and sudden gains is therefore of both scientific and clinical relevance. Such knowledge could help determine in which cases early improvement is likely and in which cases treatment planning may need to be adjusted. Although the prognostic value of early response and sudden gains for later outcome is well established, research has focused far more strongly on predicting final treatment outcome than on predicting these change patterns themselves. Overall, several research questions therefore remain unresolved. It is still unclear whether early response and sudden gains can be predicted by common factors in the therapeutic process. Moreover, patients’ subjective attributions of sudden gains have only been investigated sporadically. Furthermore, it is unclear which outcome variables are most important to patients, particularly since symptom reduction does not necessarily coincide with improved functioning (McKnight and Kashdan, 2009; Yang et al., 2022).

### 1.2 Objectives

The primary objective of the study is to identify patient-centered predictor variables of early response, sudden gains and sudden losses in outpatient psychotherapy, with the aim of enabling an earlier prognosis of treatment outcomes. Particular emphasis is placed on Grawe’s mechanisms of change at treatment onset and across the course of therapy. As a secondary aim, qualitative telephone interviews will be conducted to assess patients’ subjective attributions of change and to provide a more detailed understanding of mechanisms of change from the patient perspective. The primary hypothesis is that Grawe’s common factors, in addition to baseline symptom severity and across diagnostic categories, will explain a substantial proportion of variance in early response and sudden gains. The secondary hypothesis is that sudden gains and sudden losses in symptom severity will be only moderately associated with those alterations in psychosocial functioning and participation.

## 2 Methods

Where applicable, this study protocol was developed and reported following the updated Standard Protocol Items: Recommendations for Interventional Trials (SPIRIT) reporting guidelines (Chan et al., 2025).

### 2.1 Study design and procedure

This study uses a prospective cohort design with repeated measurements and includes both process-related and outcome-related variables. The study is observational and is conducted under naturalistic conditions. Assessment time points comprise baseline (t_0_), pre-session monitoring from sessions 1 to 27 (t_1_–t_27_), post-observation (t_28_), and a 3-month follow-up (t_29_).

All questionnaires are completed in the Greifswald Psychotherapy Navigation System (GPNS; Demir et al., 2025) using patients’ mobile devices. At the beginning of the trial sessions, patients and their therapists receive individual login credentials to access the system. Patients can complete only their assigned questionnaires and do not have direct access to diagnostic results, whereas therapists can view all questionnaire data for their own patients, including graphical progress displays. In Germany, psychotherapy is preceded by three trial sessions, which are included in the total number of sessions encompassing therapy monitoring. Following these trial sessions, 24 regular therapy sessions are incorporated into the monitoring process. Consequently, therapy monitoring is conducted across a total of 27 sessions. The monitoring consists of brief assessments repeatedly administered via the GPNS, typically immediately before each session (60 to 5 minutes before). Completion takes approximately 5 min. If a questionnaire is not completed before the session, patients may complete it shortly afterward, which is documented in the system. If patients wish to review questionnaire data during treatment, this is discussed within the therapy session. Otherwise, therapists may decide whether and how often monitoring results are discussed with patients or brought into supervision.

If a sudden gain is identified during the course of diagnostic assessment, a semi-structured telephone interview based on the Symptom Change Attribution Interview developed by Robert DeRubeis (SCAI; German et al., 2014) is initiated by the study team within 10 working days. The interview covers participants’ subjective explanations for symptom change, recalled therapeutic interventions before the sudden gain, perceived insights, behavioral changes, as well as changes in emotional experience and emotion regulation. The full interview can be found in the article by German et al. (2014). A maximum of two interviews is conducted per patient. Based on the findings of the meta-analysis of Shalom and Aderka (2020), we estimate that approximately 35% of patients will experience a sudden gain. Assuming a planned sample size of 300 patients, this would correspond to approximately 105 patients experiencing at least one sudden gain. As each patient will undergo no more than two interviews, the maximum estimated number of interviews would be 210. To ensure that interviews take place as close as possible to the event, provisional classification as a sudden gain during ongoing therapy is made without waiting for the full post-event mean comparison across the subsequent three sessions. Otherwise, the interview would be delayed by approximately 1 month. The classification of a sudden gain during ongoing psychotherapy therefore differs slightly from that used in the final analyses, while still adhering to the adapted criteria proposed by Tang and DeRubeis (1999): (a) a significant reduction in the Short Emotional Distress Inventory (SEDI; Demir et al., 2025) or the Work and Social Adjustment Scale (WSAS; Mundt et al., 2002) scores, as determined by the Reliable Change Index (RCI; Jacobson and Truax, 1991); (b) a reduction of at least 25% relative to the SEDI or WSAS score of the previous session; and (c) a significantly higher mean SEDI or WSAS score across the three sessions preceding the gain. Sudden losses are included in the quantitative analyses only; no qualitative interviews are conducted following sudden losses.

### 2.2 Study setting

Data collection takes place as part of routine outpatient psychotherapy treatment at a psychotherapy training center affiliated with the university hospital in Bielefeld, Germany. The study includes treatment from different therapeutic approaches, currently cognitive-behavioral and systemic therapy. The inclusion of additional German training clinics is being considered. The therapy sessions are conducted as individual, face-to-face and sessions at the psychotherapy training center. In exceptional circumstances, a small number of sessions may be conducted via video. Because the study is conducted under naturalistic routine-care conditions, treatment is not standardized according to a single treatment manual or fixed session protocol. Instead, the interventions are individualized based on the treatment needs and therapeutic progress of each patient. This design reflects routine outpatient psychotherapy, in which treatment is delivered under ordinary clinical-care conditions rather than according to a research-imposed intervention protocol. The total number of treatment sessions follows German regulatory standards. Typically, treatment comprises 12–60 sessions; in exceptional cases, treatment may extend to a maximum of 80 sessions. In addition, four initial sessions (“probatory sessions”) take place at the beginning of treatment. Each session lasts 50 min. Sessions are most often scheduled on a weekly basis. However, deviations from this schedule may occur in accordance with clinical requirements.

All therapists hold a master’s degree in psychology and are in advanced postgraduate training to become licensed psychotherapists. Before commencing clinical work at the psychotherapy training center, therapists have usually acquired at least 18 months of professional experience in psychiatric and/or psychosomatic clinics. Therapists are required to discuss their cases with an experienced supervisor, typically every four sessions. Treatment adherence is therefore ensured by the therapist’s advanced level of training and the provision of structured clinical supervision.

### 2.3 Participants and eligibility criteria

The study includes all individuals who begin outpatient psychotherapy at the participating training center and provide informed consent for study participation. Participants do not receive financial compensation or other incentives for their participation. Licensed psychotherapists at the training center determine during the initial consultation whether exclusion criteria for outpatient psychotherapy apply. These institutional exclusion criteria are therefore also reflected in the study sample. Exclusion criteria:

- No statutory health insurance.
- Age under 18 years.
- Outpatient psychotherapy within the previous 2 years.
- Addictive behavior without motivation for abstinence.
- Acute psychiatric situations requiring inpatient treatment, including acute suicidality without the ability to make a credible no-suicide pact, acute manic or psychotic episodes, or life-threatening anorexic states.
- Multiple previous suicide attempts.
- External motivation without personal concerns or without subjective psychological distress.
- Absence of a mental disorder meeting clinical criteria.
- Ongoing criminal proceedings.

The training center primarily treats affective disorders, these are, therefore, expected to represent the largest diagnostic group in the sample. Nevertheless, the study aims to include patients from as many diagnostic groups as possible.

### 2.4 Outcomes

The primary outcome measures are sudden gains in symptom distress measured by the SEDI and sudden gains in daily functioning measured by WSAS. Sudden gains are defined according to established, adapted criteria based on Tang and DeRubeis (1999): (a) a significant reduction of the SEDI score or the WSAS score calculated based on the RCI (Jacobson and Truax, 1991), (b) a reduction of at least 25% relative to the SEDI or WSAS score from the previous session, (c) a significantly higher mean SEDI or WSAS score across the three sessions before the gain than across the three sessions after the gain.

Early response is examined as an additional primary outcome and is defined as reliable significant change in symptom severity and/or daily functioning up to and including session 5, calculated using the RCI for the SEDI and the WSAS. Sudden losses are also examined and are defined analogously to sudden gains, with increases rather than decreases in SEDI or WSAS scores.

### 2.5 Predictors

Grawe’s general mechanisms of change are assessed primarily using the Bern Post Session Report/Patient Questionnaire (BPSTB-2000; Flückiger et al., 2010), while the Intersession Processes measure (ISP; Demir et al., in preparation), Alliance (Demir et al., 2025), Insight (Demir et al., 2025), and Alliance Rupture and Repair Screening (R/R-S, Demir et al., in preparation) are used as supplementary process measures. To assess patients’ expectations regarding therapy and the therapeutic relationship, modified versions of the BPSTB-2000, Alliance and Insight are used. These modified versions retain the original items but are minimally reworded (e.g., shifted to future tense or prefaced with “I expect that…”) so that they assess expectations about the constructs that will later be measured repeatedly during treatment.

### 2.6 Data collection plan and measurements

Data are collected using the GPNS as a digital therapy monitoring tool. All questionnaires are transdiagnostic self-report measures. Table 1 provides an overview of the assessment schedule and outcome collection.

**Table 1:** Schedule of enrolment, interventions, and assessments.

Timepoint	Study period				
	Registration	Baseline	Monitoring	Post (end of observation period)	Follow-up after 3 months
	−t_1_	t_0_	t_1_–t_27_	t_28_	t_29_
Enrolment					
Study information and informed consent	x				
Sociodemographic data		x			
Treatment history		x			
Interventions					
Outpatient psychotherapy (treatment as usual)			x		
Assessments: outcomes					
SEDI		x	x	x	x
WSAS		x	x	x	x
Assessments: predictors					
BPSTB-2000		x (mod.)	x		
GROC			x		
ISP			x		
Alliance		x (mod.)	x		
R/R-S			x		
Insight/Understanding		x (mod.)	x		
Exploratory data collection					
Qualitative phone interview on perceived reasons for change			x (after sudden gain)		

Alliance, therapeutic relationship. BPSTB-2000, Bern Patient Session Questionnaire 2000; GROC, Global Rating of Change Scale; Insight/Understanding, Insight and Understanding Scale; ISP, Intersession Processes Scale; Modified Alliance, expectations of the therapeutic relationship; Modified BPSTB-2000, Modified version of the Bern Patient Session Questionnaire; Modified Insight/Understanding, expected insight and understanding; R/R-S, rupture/repair screening. SEDI, Short Emotional Disorder Inventory; WSAS, Work and Social Adjustment Scale.

At baseline (t_0_), sociodemographic information, medication, and treatment history are assessed. In addition, baseline assessment includes the modified BPSTB-2000 (patient version; 22 items, 7-point Likert scale) to assess expectations regarding therapeutic processes, the modified questionnaire on expectations regarding the therapeutic relationship (Alliance; 8 items, 4-point Likert scale), the modified questionnaire on expectations regarding insight (Insight; 9 items, 4-point Likert scale). Baseline assessment also includes the SEDI (15 items, 4-point Likert scale) to assess general symptom severity and the WSAS (5 items, 8-point Likert scale; adapted German translation) to assess impairments in functioning due to mental health problems. The SEDI and WSAS are administered again at the other main assessment points, i.e., the post-observation period (t_28_) and the 3-month follow-up (t_29_). At the post-observation period (t_28_), participants are also asked whether any changes in their medication occurred during the course of psychotherapy. If applicable, they are asked to specify the nature of these changes and the time at which they exactly occurred.

Before each therapy session (t_1_–t_27_), the following instruments are administered as part of therapy monitoring: SEDI, WSAS, BPSTB-2000, Global Rating of Change Scale (GROC; 1 item, 11-point Likert scale; Kamper et al., 2009) for global self-assessment of change, the ISP (7–13 items, dichotomous scale and 5-point Likert scale) to assess therapy-related activities between sessions, as well as the Alliance questionnaire supplemented by the Alliance Rupture and Repair Screening (1–2 items, 4-point & 5-point Likert scale) to identify and assess discrepancies in the therapeutic relationship and their resolution, as well as the Insight questionnaire (Insight; 9 items, 4-point Likert scale), assessing patients’ understanding of conditions contributing to and maintaining mental health problems.

### 2.7 Interventions and concomitant care

Participants receive routine outpatient psychotherapy in accordance with standard clinical practice as treatment as usual (either CBT or systemic therapy). Additional psychiatric treatment and pharmacotherapy are permitted. During day clinic or inpatient treatment, outpatient psychotherapy and study data collection are paused and resumed after discharge. No further restrictions are imposed regarding concomitant care.

### 2.8 Harms

No significant harm is expected as a result of participating in the study. Although topics covered in the interview may cause temporary discomfort, this is considered unlikely and is not expected to exceed the level of stress encountered in everyday life.

### 2.9 Participant timeline

Participant recruitment is planned from approximately September 2026 to June 2028. The study completion is scheduled for March 2029.

### 2.10 Recruitment

Participants are recruited during the initial consultation at the participating psychotherapy center. The initial consultation takes place before the start of the outpatient therapy and is conducted by licensed psychotherapists who have previously received training from the project team on how to present the information in a structured, transparent, and comprehensible manner, thereby enabling participants to make an informed decision. This standardized procedure is intended to support a high participation rate while ensuring that participation remains voluntary.

### 2.11 Sample size estimation

Sample size was determined *a priori* using Monte Carlo simulation for a generalized linear mixed-effects model (GLMM) with repeated weekly measurements (Level 1: week/measurement time point; Level 2: person). A closed-form, generally accepted analytical power formula for complex GLMM structures (particularly those with time-varying predictors and random effects) is only available to a limited extent; therefore, power was determined using simulation-based methods.

The primary endpoints are binary: early response as a reliable improvement after five treatment sessions (yes/no), occurrence of a sudden gain (yes/no; modeled as a weekly event or event probability over the weeks) and occurrence of a sudden loss (yes/no; modeled as a weekly event or event probability over the weeks). The planned GLMM examines whether weekly predictors, particularly Grawe’s common factors such as the therapeutic alliance, predict the probability of early response or sudden gains while accounting for interindividual variability through random intercepts and, where appropriate, random slopes. Effect size assumptions were primarily based on Wucherpfennig et al. (2017), who reported small to moderate effects for changes in common factors in the context of sudden gains (e.g., therapeutic alliance: *d* ≈ 0.27; coping skills: *d* ≈ 0.50 in critical sessions/phases). Because the planned analyses include logistic models, these effects were translated conservatively into small to moderate odds ratios, typically in the range of approximately 1.3 to 1.6 per standard deviation increase in a predictor, depending on scaling. It was also assumed that partial effects in multivariable models would be smaller than bivariate effects.

The rate of early response after five sessions was estimated at approximately 48%, based on a meta-analysis by Beard and Delgadillo (2019). The frequency of sudden-gain events was assumed to be approximately 35% based on the meta-analysis by Shalom and Aderka (2020). These values served as empirically grounded initial parameters for generating the simulated datasets. The planned models include several common-factor predictors, relevant covariates such as baseline symptom severity, time effects, and selected interactions. Since longitudinal psychotherapy studies are likely to have incomplete measurement series, a dropout or missingness buffer of approximately 10–20% was incorporated. Within the framework of the Monte Carlo simulations, synthetic datasets were repeatedly generated under these assumptions, analyzed using the planned generalized linear mixed-effects model (GLMM), and the significance of the central fixed effect was tested at an α level of 0.05. The simulations showed that a sample of *n* = 300 individuals provides sufficient statistical power of at least 80% to detect small to moderate effects of the common factors on the probability of an early response or sudden gains.

### 2.12 Data collection plan: retention

Several measures are implemented to ensure data quality, including training procedures, standardization of data collection, and strategies to reduce missing data. The licensed therapists conducting the informed consent process receive specialized and standardized training on the study information: this includes a transparent presentation of the study’s content, handling of questions, and objective, non-coercive encouragement to participate. The goal is to ensure consistent, high-quality information for all patients while guaranteeing the voluntary nature of participation. Psychotherapists in training also receive detailed information on the proper integration of the GPNS into practice routines and the use of monitoring data for progress assessment. This is intended to ensure that the surveys are conducted consistently and that the collected data are appropriately integrated into the therapeutic process. To reduce missing data, therapists are instructed to actively ask at the beginning of each session whether the designated GPNS questionnaires have been completed. This allows for any missing entries to be identified immediately and, where possible, corrected. The use of the centralized digital monitoring system ensures standardized data collection with regard to the timing of the survey, the order of items, and data storage. Overall, these measures help to ensure the validity, completeness, and comparability of the collected data.

### 2.13 Data management

Data are stored in pseudonymized form using unique identification numbers. Person-identifying information is stored separately from research data. Electronic data is stored on secure, password-protected servers of the training clinic. Data entry is conducted digitally via the GPNS. Missing values are automatically detected by the system. Data entry is digital, and missing values are automatically flagged by the system. Data processing is conducted in accordance with the data protection regulations of the healthcare institution. Study documentation forms part of the patient file and must be made available to patients upon request. After data collection is completed, therapists will print the data history and file it in the paper record at the training clinic. All study data will be anonymized as soon as possible after completion of data collection, and personal data will be deleted by the study team no later than at the end of the study. Therapists will then no longer have digital access to the data.

### 2.14 Statistics

#### 2.14.1 Statistical analysis outcomes

The statistical analysis is hypothesis-driven and primarily relies on generalized linear mixed-effects models to account for the hierarchical data structure with repeated measurements (level 1: week/measurement time point) within individuals (level 2: individual). Models will include a random intercept for each patient but no random slopes. Nonlinear associations between continuous predictors and outcomes will be examined by adding quadratic or spline-based terms to the fixed part of the model. Final model selection will be based on the Bayesian Information Criterion (BIC). The primary endpoints are binary: (1) early response (yes/ no), (2) occurrence of a sudden gain (yes/ no) and (3) occurrence of a sudden loss (yes/ no), modeled either as a weekly event or as an event probability across the observation period. For both endpoints, logistic mixed-effects models with at least a random intercept at the patient level will beapplied. Random slopes will be tested and retained only if empirically justified, for example by improved model fit, convergence, and interpretable variance components.

To test the main hypothesis, multivariable models will include Grawe’s common factors (assessed weekly using the BPSTB-2000, ISP, Alliance, R/R-S, Insight) as time-varying predictors, alongside baseline assessments of general therapy expectations and expectations regarding the therapeutic relationship (assessed using the modified BPSTB-2000, modified Alliance, modified Insight), entered as single-assessment predictors measured once. Baseline symptom severity (SEDI), diagnoses, baseline functioning (WSAS), sociodemographics, previous treatment experience, medication status and medication changes during treatment (yes/no) will be entered as covariates for adjusted estimation. Effects will be reported as odds ratios with 95% confidence intervals; inferential statistical tests are two-sided at *α* = 0.05. Alternative specifications, including different time functions and nonlinear terms for continuous predictors, will be evaluated using appropriate fit indices and likelihood-based methods. Baseline symptom severity and diagnoses will also be included as a potential moderator to test whether the association between common factors and outcomes varies across levels of baseline severity, as well as therapy type (behavioral vs. systemic). All moderation analyses are labeled secondary and will be reported transparently, including effect sizes, confidence intervals, and complete model outputs. To test the secondary hypothesis, which posits that sudden gains or losses in symptom severity are only moderately associated with changes in psychosocial functioning, and to address the potentially time-varying and reversible association between symptoms and functioning, we will jointly model weekly SEDI and WSAS scores in bivariate mixed-effects models with a bivariate normally distributed random effect to capture potential correlations. Consistent with the primary models, these bivariate models will include correlated random intercepts only; random slopes will be omitted. Associations between symptom and functioning events will be examined via correlations of event indicators and mixed-effects models that compare weeks with different combinations of symptom and functioning events. To examine the robustness of the primary findings, we will conduct sensitivity analyses in which we re-estimate all primary logistic mixed-effects models using alternative operationalizations of the binary outcome events. For sudden gains and losses, we will apply a modified version of the stability criterion (criterion 3) according to Tang and DeRubeis (1999), allowing the comparison of two sessions before and after the gain instead of three when three sessions are not available or when applying the original criterion would exclude early or late gains. For early response, we will use the same reliable change index criterion but restrict the time window from session 5 to session 4 to test a more conservative definition of early response. The direction, magnitude, and statistical significance of effects from these sensitivity analyses will be compared with those from the primary analyses.

Telephone interviews will be analyzed in MAXQDA using qualitative content analysis. Transcribed patient responses will be coded by the project team, and a hierarchical category system will be developed inductively to identify recurring content patterns and broader subjective mechanisms of change.

#### 2.14.2 Statistical analysis population and missing data

All enrolled patients with at least one evaluable post-baseline assessment are included in primary analyses for early response, regardless of treatment discontinuation, irregular attendance, or protocol deviations. For sudden gains and losses analyses, at least seven assessments are required to reliably detect abrupt changes. Dropout is not an exclusion criterion, the focus is on whether patients have sufficient data to assess early response, sudden gains or sudden losses prior to dropout. This reflects an intention-to-treat-like approach, distinct from the classical definition, intentionally incorporating all available data to mirror routine clinical practice. Mixed-effects models (including growth mixture models) are used, which robustly handle missing data under a missing-at-random assumption by utilizing all available observations and accommodating unbalanced measurement series (patients do not drop out listwise due to incomplete data) (Enders, 2010).

Sensitivity analyses examine robustness across different missing-data patterns, particularly when missingness may be systematic. If imputation is needed, predictors of missingness (e.g., baseline symptom severity, prior illness course, demographics, earlier monitoring participation) will be included. The final report will transparently describe the extent, timing, and reasons (where available) for missing data, along with comparisons of key baseline characteristics between complete and incomplete datasets. Given our observational focus on naturalistic treatment trajectories and abrupt changes rather than comparative efficacy of specific therapies, inclusion of all available data is more critical than strict per-protocol criteria.

### 2.15 Monitoring

While data collection is ongoing, an interim statistical analysis of sudden gains will be conducted, whereas all remaining analyses will take place after completion of data collection. Only the study team has access to interim results. Specifically, only sudden gains identified in individual patients will be communicated back to those patients within the qualitative interview procedure. Problems (for example regarding the GPNS system) and questions may be reported to the study team by therapists and patients via a weekly telephone consultation hour or by email. All reported difficulties will be documented systematically in a shared document, and the study team will continuously assess whether protocol modifications are necessary. Discontinuation of the study would occur if a participant explicitly requested to withdraw or if outpatient treatment - and consequently participation in the study—could not be continued due to adverse events. These adverse events include the participant’s death or circumstances such as acute suicidality or an acute risk of harm to others requiring immediate crisis intervention, as well as any other circumstances that would preclude the continuation of outpatient treatment. The treating therapists is responsible for monitoring of adverse events, not the study team. For reasons of professional confidentiality, these events may be communicated to the study team only by the participants themselves and only if they wish to do so. Consequently, systematic assessment of such events by the study team is limited and cannot be assumed to be complete. If serious unforeseen events require early termination of the study, this decision will be made by the study director (CS).

## 3 Discussion

This study is one of the first to examine therapy process variables quantitatively as predictors of sudden gains, sudden losses and early response while also incorporating complementary qualitative methods. Previous qualitative work on mechanisms of sudden gains has generally relied on small samples sizes (Adler et al., 2013; Goodridge and Hardy, 2009; Lemmens et al., 2021) or on retrospective data collected months or years after the event, which limits the availability of detailed event-related information (Davies et al., 2006). In contrast, telephone interviews conducted shortly after sudden gains may facilitate more accurate recall of relevant processes and may also promote patients’ awareness of adaptive change patterns that could further support psychotherapy. A potential limitation of conducting interviews during ongoing treatment is that they may affect the treatment by directing patients’ attention to therapeutic change and prompting them to reflect more deeply on the experiences and mechanisms underlying this change. Such reflection may involve the development of insight into the associations between interpersonal patterns, emotional experiences, and psychological symptoms and also adopting different perspectives. This possibility is consistent with previous research suggesting that a greater flexibility in shifting between immersed and distanced perspectives when processing emotional experiences may be associated with subsequent symptom change and therapeutic progress (Barbosa et al., 2021). Moreover, a meta-analysis found a moderate association between patient insight and psychotherapy outcome across treatment modalities (Jennissen et al., 2018). These findings suggest that the reflective processes elicited during an interview could, in principle, support subsequent symptom change or therapeutic progress. However, without the interviews, no insights into subjective mechanisms of change can be obtained, and we therefore accept this limitation.

A related consideration applies to the provision of feedback based on routine outcome monitoring. Providing progress feedback can itself be regarded as a potentially therapeutic intervention, as it makes patients’ and therapists’ perceptions of treatment progress and difficulties more explicit and may prompt adjustments to the ongoing therapeutic process. As demonstrated by a meta-analysis, progress feedback may have a beneficial effect on treatment outcomes, although the observed effect sizes were small (de Jong et al., 2021). Thus, providing feedback during treatment may introduce an additional source of influence on subsequent therapeutic processes and outcomes, and may therefore constitute a potential confound when interpreting treatment courses. Nevertheless, the present study was deliberately designed to reflect the way in which routine outcome monitoring is used in naturalistic clinical care. Feedback was therefore not restricted to the end of treatment but was provided during treatment at the discretion of patients and therapists. This approach reflects the clinical purpose of monitoring: it enables both parties to identify progress and emerging difficulties in a timely manner and creates opportunities to adapt the ongoing therapeutic process where necessary. Restricting feedback until the end of treatment would consequently have reduced the ecological validity of the protocol and would not have represented the intended use of routine outcome monitoring in routine care. We therefore retained feedback during ongoing treatment despite the possibility that it may influence, and potentially confound, subsequent therapeutic processes and outcomes.

By examining early response, sudden gains and losses within a single design, the study further enables the identification of shared and distinct predictors of different change patterns, thereby contributing to a more differentiated understanding of psychotherapeutic change processes. Given the established importance of early response, sudden gains and losses for treatment outcome, a better understanding of the conditions under which these changes occur appears clinically relevant (Beard and Delgadillo, 2019; Röhrig et al., 2024; Shalom and Aderka, 2020). The anticipated findings may contribute to a more differentiated understanding of psychotherapeutic change processes and to improved prediction of treatment trajectories. They may also help identify practical ways of structuring psychotherapy so that conditions conducive to early response and sudden gains are more likely to emerge.

Conducting the study in a naturalistic setting allows conclusions to be drawn under routine outpatient conditions. At the same time, these conditions entail greater procedural variability than highly controlled trials. For example, session frequency and session length may vary, double sessions may occur, or therapists may be absent for extended periods. Such variability may complicate regular close-meshed monitoring and may limit patients’ ability to recall session-specific content relevant to symptom change when longer intervals occur between sessions. Another challenge involves potential structural changes at the training center, including adjustments related to statutory training reform. These changes could reduce the number of available therapy spots. This could hinder the achievement of the planned sample size. For this reason, the inclusion of additional training clinics is being considered. A further limitation is that outcomes are assessed exclusively by patient self-report. Therapist ratings are not collected in order to minimize the workload. However, self-reports may be affected by disorder-specific response tendencies, such as negatively biased self-evaluation in depressive disorders or more extreme response styles in borderline personality disorder (Everaert et al., 2017; Kaufman et al., 2023). Moreover, awareness that therapists can access questionnaire results may increase socially desirable responding (Krumpal, 2013). To minimize bias, both digital surveys and interviews use wording that is intended to be as neutral and non-suggestive as possible, and patients are encouraged within the GPNS to respond realistically and intuitively. To enhance feasibility and facilitate potential implementation in routine outpatient therapy beyond the scope of the study, we use comparatively brief questionnaires.

### 3.1 Ethics and dissemination

#### 3.1.1 Research ethics approval

The study was approved by the Bielefeld Ethics Committee (number EUB-2026-046-W1) and registered in the German Clinical Trials Registry (registration number DRKS00039264). The study is conducted in accordance with the Guidelines for Safeguarding Good Research Practice of the German Research Foundation (German Research Foundation, 2025).

#### 3.1.2 Protocol amendments

Any relevant amendments affecting study conduct or evaluation will be documented on the DRKS study page (https://www.drks.de/search/de/trial/DRKS00039264), where the history of changes can also be reviewed. If new findings emerge that may affect participants’ willingness to continue in the study, participants will be informed accordingly.

#### 3.1.3 Consent or assent

All participants will receive comprehensive verbal and written information about the study’s objectives, content, and procedures, as well as data protection and data handling. Written informed consent will be obtained before participation. Consent to participate in the study is not a prerequisite for receiving outpatient psychotherapy at the training institute.

#### 3.1.4 Confidentiality and data access

Personal data will be collected and processed as part of the study. Data will be stored and analyzed in pseudonymized form. Direct access is restricted to authorized members of the research team and to therapists with regard to their own patients. Supervisors do not have direct access, although pseudonymized data may be discussed in supervision. After completion of data collection, all data will be anonymized as soon as possible, and all personal data will be deleted no later than at the end of the study. After study completion, anonymized research data will be made available to third parties only within the restrictions required by data protection law, for example through secure online transfer or on-site access in a research data center based on individual contractual agreements. Research data will be published only in a form that does not permit identification of individual participants and will be shared on reasonable request, e.g. for a meta-analysis. In doing so, the study follows German Research Foundation guidance on good scientific practice (German Research Foundation, 2025).
